# Spin-current-mediated rapid magnon localisation and coalescence after ultrafast optical pumping of ferrimagnetic alloys

**DOI:** 10.1038/s41467-019-09577-0

**Published:** 2019-04-15

**Authors:** E. Iacocca, T.-M. Liu, A. H. Reid, Z. Fu, S. Ruta, P. W. Granitzka, E. Jal, S. Bonetti, A. X. Gray, C. E. Graves, R. Kukreja, Z. Chen, D. J. Higley, T. Chase, L. Le Guyader, K. Hirsch, H. Ohldag, W. F. Schlotter, G. L. Dakovski, G. Coslovich, M. C. Hoffmann, S. Carron, A. Tsukamoto, A. Kirilyuk, A. V. Kimel, Th. Rasing, J. Stöhr, R. F. L. Evans, T. Ostler, R. W. Chantrell, M. A. Hoefer, T. J. Silva, H. A. Dürr

**Affiliations:** 1 Department of Applied Mathematics, University of Colorado, Boulder, CO 80309 USA; 2 National Institute of Standards and Technology, Boulder, CO 80305 USA; 3 Department of Physics, Division for Theoretical Physics, Chalmers University of Technology, Gothenburg, 412 96 Sweden; 4 SLAC National Accelerator Laboratory, 2575 Sand Hill Road, Menlo Park, CA 94025 USA; 5 School of Physics, Science, and Engineering, Tongji University, Shanghai, 200092 China; 6 Department of Physics, University of York, York, YO10 5DD UK; 7 Department of Physics, Stockholm University, Stockholm, 106 91 Sweden; 8 Department of Molecular Science and Nanosystems, Ca’ Foscari University of Venice, Venezia-Mestre, 30172 Italy; 9 Department of Physics, Temple University, 1925 N. 12th St., Philadelphia, PA 19122 USA; 10 Spectroscopy & Coherent Scattering, European X-Ray Free-Electron Laser Facility GmbH, Holzkoppel 4, 22869 Schenefeld, Germany; 11 Department of Electronics and Computer Science, Nihon University, 7-24-1 Narashino-dai Funabashi, Chiba, 274-8501 Japan; 12 Institute for Molecules and Materials, Radboud University, Heyendaalseweg 135, 6525 AJ Nijmegen, The Netherlands; 13 Physique des Matériaux et Nanostructures, Université de Liège, Liège, B-4000 Sart Tilman Belgium; 14 Faculty of Arts, Computing, Engineering and Sciences, Sheffield Hallam University, Howard Street, Sheffield, S1 1WB UK; 15 Department of Physics and Astronomy, Uppsala University, Box 516, 751 20 Uppsala, Sweden

## Abstract

Sub-picosecond magnetisation manipulation via femtosecond optical pumping has attracted wide attention ever since its original discovery in 1996. However, the spatial evolution of the magnetisation is not yet well understood, in part due to the difficulty in experimentally probing such rapid dynamics. Here, we find evidence of a universal rapid magnetic order recovery in ferrimagnets with perpendicular magnetic anisotropy via nonlinear magnon processes. We identify magnon localisation and coalescence processes, whereby localised magnetic textures nucleate and subsequently interact and grow in accordance with a power law formalism. A hydrodynamic representation of the numerical simulations indicates that the appearance of noncollinear magnetisation via optical pumping establishes exchange-mediated spin currents with an equivalent 100% spin polarised charge current density of 10^7^ A cm^−2^. Such large spin currents precipitate rapid recovery of magnetic order after optical pumping. The magnon processes discussed here provide new insights for the stabilization of desired meta-stable states.

## Abstract (web summary)

The understanding of the magnetisation evolution upon femtosecond optical pumping remains elusive. The authors perform resonant X-ray magnetic scattering measurements and multiscale simulations that reveal rapid magnetic order recovery in ferrimagnets via nonlinear magnon processes.

## Introduction

Spin dynamics upon femtosecond optical pumping^1–15^ have been intensely studied during the last two decades both because of potential applications for information storage and the need to understand the fundamental physics involved^16^. A variant of these dynamics is all-optical switching (AOS). While originally demonstrated for ferrimagnetic alloys with perpendicular magnetic anisotropy (PMA)^2^, AOS has been reported to occur in ferromagnetic PMA materials subject to optical pumping^9–12^ or by use of ultrafast hot electrons^14,15,17^. Whereas the picosecond magnetisation dynamics, even for non-uniform states^5,6^, has been successfully modelled with spatially averaged, quasi-equilibrium models^1,3,4,18^, there is a growing understanding of the important role played by spatially-varying magnetisation. For example, the chemical inhomogeneity of amorphous ferrimagnetic GdFeCo alloys results in the picosecond transfer of angular momentum that both drives magnetisation switching^8^ and influences the equilibrium state after pumping with a single laser pulse^13^. More recently, the effective domain size during cooling has been identified as a criterion to predict whether macroscopic AOS can occur^12^.

To further investigate the fundamental physics involved in the evolution of spatially varying magnetisation after ultrafast optical pumping, and to elucidate which physical mechanisms are most important for the recovery of local magnetic order at picosecond timescales, we study the space- and time-dependent magnetisation dynamics in ferrimagnetic Gd_0.24_Fe_0.665_Co_0.095_ alloys with time-resolved resonant X-ray scattering. We then compare our data with a multiscale model that utilises both atomistic and large-scale micromagnetic components to simulate the spatiotemporal evolution of the magnetisation. We identify two distinct dynamic processes, which we term magnon localisation and magnon coalescence. These processes describe the nucleation and subsequent dynamics of localised textures that arise from attractive nonlinear interactions between thermalized magnons^19^. This is in contrast to theories that predict the order parameter recovery of the spatially averaged magnetisation, as described by the damping of a heated spin-wave distribution^20^.

Magnon localisation is the process by which small, non-equilibrium, localised magnetic textures nucleate and grow. The textures are necessarily long-term unstable transient features that are not to be confused with magnetic domains, which are equilibrium or meta-stable states. In the context of conservative dynamics, localised textures can be described as dynamical magnon bound states^21^ known as magnon drops^22^. Magnon localisation can be detected by the appearance of a broad ring centred at low *q* in the X-ray scattering pattern with a rapid radius expansion in reciprocal space and simultaneously decreasing ring width. A subsequent shrinking of the ring radius accompanied by the continual decrease in the ring width indicates a stage of magnon drop growth we term magnon coalescence. Microscopically, magnon coalescence is driven by the ongoing nonlinear attraction between magnon drops and unbound magnons that depletes the thermal magnon population as the magnon drops continue to grow, on average. The substantial, highly turbulent flux of angular momentum in the vicinity of magnon drops during the coalescence stage can be estimated by use of numerical simulations accompanied by a hydrodynamic formulation of magnetisation dynamics that show the presence of strong exchange flow spin currents (EFSCs)^23,24^, which are equivalent to a 100% spin polarised charge current density on the order of 10^7^ A cm^−2^. These simulation results suggest that magnon drop dynamics driven by such large EFSCs expedite magnon coalescence via magnon drop growth, break-up, and merger^25^.

Our study suggests that the picosecond evolution of the spatially varying magnetisation can be understood from a phase kinetics approach^26,27^. When the magnetisation quenching upon femtosecond optical pumping is almost 100%, the initial condition of the system can be described as a non-equilibrium distribution of randomised spins that undergo rapid restoration of the magnetic order parameter, subject to a multiplicity of final equilibrium (or quasi-equilibrium) states. In other words, the subsequent rapid passage from a nearly paramagnetic to a magnetically ordered state will generally do so via pathways of unstable magnon-drop growth, i.e., phase-ordering kinetics. Such dynamics are in contrast to the critical behaviour expected from an adiabatic evolution through a phase transition^28^. Because of the large degeneracy of the equilibrium states, unstable growth necessarily leads to pattern formation, examples of which include domains in magnetic materials^29^ and metallic alloys^26^, phase separation in binary fluids and superfluids^30^, and optical solitons^31^. In addition, it has been argued that rapid quenching of a randomised state can dynamically stabilise topological defects via the Kibble–Zurek mechanism^32,33^, as seen in superfluids^30,34^, ferroelectrics^35^, magnetic vortices^36^, and bubble domain lattices^37^. Therefore, the magnon processes identified here shed light upon the physical mechanisms that are important in the initial stages of unstable growth and pattern formation triggered by ultrafast optical pumping.

## Results

### X-ray scattering

**Fig. 1:**
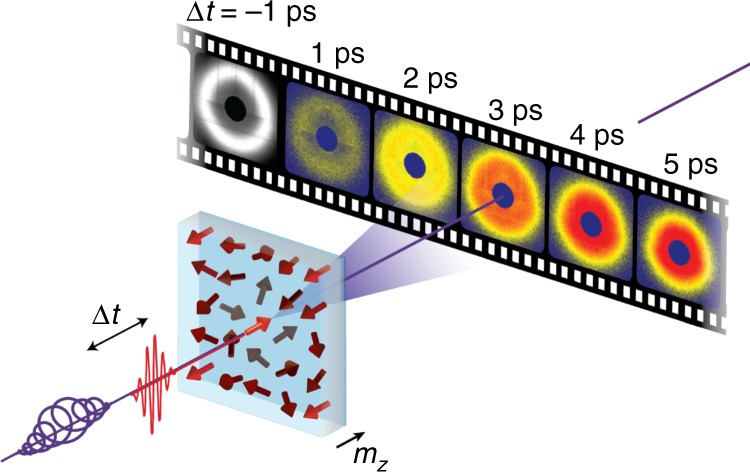
Schematic of the experimental setup. A femtosecond optical pulse randomises the spin degree of freedom and a subsequent circularly polarised X-ray pulse probes the perpendicular magnetisation, *m*_*z*_, at a given delay, Δ*t*. For each time delay, the two-dimensional X-ray scattering intensity map is obtained, from which the spin–spin correlation function can be extracted. X-ray magnetic circular dichroism is simultaneously measured by the un-scattered beam

The evolution of the scattered intensity is measured by time-resolved, resonant magnetic soft X-ray scattering, a pump-probe technique schematically shown in Fig. 1 (see details in Methods). A 0.5 T field is applied perpendicular to the film plane during the measurement. As such, the magnetisation is always reset into the saturated state prior to each pump pulse. The element-specific, spatially-averaged dynamics are simultaneously measured by X-ray magnetic circular dichroism (XMCD) of the unscattered beam.

From the scattered intensity measurements, we directly obtain the time-varying spin–spin correlation function, Δ*S*^2^(*q,t*) (see Methods). This quantity provides information related only to the magnetisation’s spatial profile, in contrast to the spin correlations projected onto the sample’s chemical nanostructure studied in ref. ^8^. The background spin–spin correlation signal prior to time-zero is an order of magnitude smaller than the features observed at *t* > 0.

**Fig. 2:**
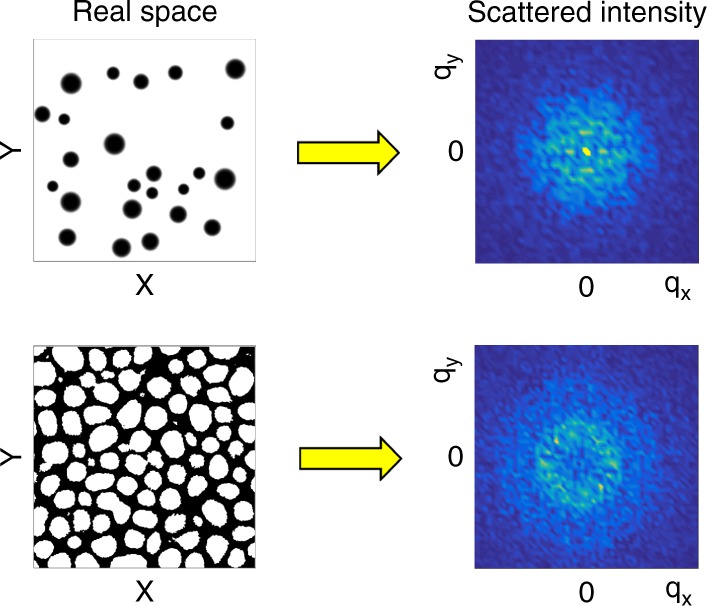
Schematic examples of scattered intensities from real-space features. The left column shows real-space patterns while the right column shows the corresponding scattered intensity computed via Fourier transform with colour scale in arbitrary units. In the top row, a random distribution of circular, localised textures gives rise to a broad feature centred at *q* = 0 in the scattered intensity. In the bottom row, localised textures possessing long-range correlations result in a ring pattern in the scattered intensity

For illustrative purposes, we show two schematic examples in Fig. 2 as to the expected scattering patterns correlated to representative spatial patterns. A broad peak centred at *q* = 0 corresponds to a low density of randomly located textures of variable size^38^, as schematically shown in the top row. However, if these azimuthally disordered textures are sufficiently close-packed so as to have a well-defined averaged spatial separation, they will exhibit a long-range correlation length^39^, i.e., a ring structure develops, as shown in the bottom row.

**Fig. 3:**
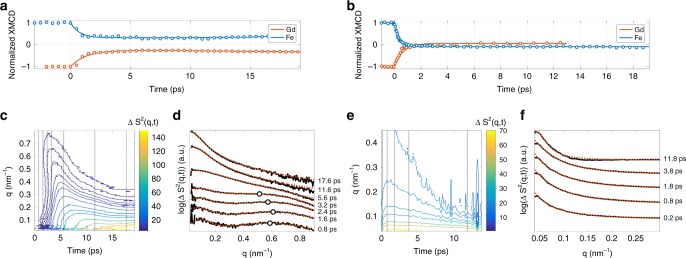
Experimental XMCD and spin–spin correlation. XMCD data is shown in **a** non-AOS obtained in a 30 nm-thick sample subject to an absorbed fluence of 3.91 mJ cm^−2^ and **b** for AOS obtained in a 20 nm-thick sample subject to an absorbed fluence of 4.39 mJ cm^−2^. Solid lines are guides to the eye. **c** Contours of the azimuthally averaged spin–spin correlation function, Δ*S*^2^(*q*,*t*), for non-AOS. For the time instances indicated by dotted vertical lines, lineouts are shown by black curves in (**d**) and are vertically shifted for clarity. Fits to the data with a Lorentzian line-shape for the low-*q* diffraction ring below *q* = 0.1 nm^−1^ and a Gaussian line-shape for the high-*q* diffraction ring above *q* = 0.4 nm^−1^ are shown by dashed red curves. The black circles indicate the fitted ring radius of the Gaussian component. **e** Contours of the azimuthally averaged spin–spin correlation function, Δ*S*^2^(*q*,*t*), for AOS. For the time instances indicated by dotted vertical lines, lineouts are shown by black curves in (**f**) and are also vertically shifted for clarity. Fits to the data with a Lorentzian line-shape are shown by dashed red curves

We measured the magnetisation dynamics for two cases where the XMCD data within 20 ps exhibits partial or full quench of the Gd and Fe moments. We refer to non-AOS for partial quench and AOS for full quench. As shown below, the dynamic scattering data are qualitatively similar between these cases. We reiterate that the applied field saturates the sample so that any large amplitude inhomogeneity in the spatial spin distribution is not stable at long times. Non-AOS was obtained with a 30 nm thick sample and an absorbed 800 nm pump fluence of 3.91 mJ cm^−2^. In Fig. 3a, the corresponding XMCD response for both Gd and Fe exhibits a partial quench of the magnetisation for *t* < 3 ps, followed by an approximately constant state of demagnetisation up to the longest delay time of 20 ps. AOS was not achieved with the available pump fluences in this sample. Using a 20 nm thick sample and an absorbed 800 nm pump fluence of 4.39 mJ cm^−2^, AOS was achieved. The XMCD data in this case shows that the magnetic moments are fully quenched and switch at ≈3 ps, as presented in Fig. 3b. However, similar to the non-AOS case, the spatially averaged magnetisation remains approximately constant for as long as 20 ps after time-zero. The extremely slow time dependence of the XMCD data in both cases indicates that the average magnetisation is essentially constant for 3 ps < *t* < 20 ps. A critical implication is that the quasi-thermal redistribution of magnon occupation caused by either damping or other inelastic interactions that eventually drives the magnetisation towards a saturated state is not important at these timescales.

The azimuthally averaged spin–spin correlation function for Gd in the non-AOS case is shown by contours in Fig. 3c. Spin–spin correlation profiles at selected time instances are shown in Fig. 3d by solid black curves that have been shifted vertically for clarity. These lineouts have two ring-like spectral features: one with a radius close to or below the smallest resolved wavevectors and one with a radius in the range 0.4 nm^−1^ < *q* < 0.8 nm^−1^. Fits to the data shown by the dashed red curves are obtained by using a Gaussian line-shape for the high-*q* feature (with a ring radius indicated by black circles) and a Lorentzian line-shape for the low-*q* feature. The fitted Gaussian line-shape indicates the appearance of a ring and therefore a short-range correlated magnetisation pattern at sub-picosecond timescales. After ≈5 ps, reliable fits were obtained by use of only a Lorentzian line-shape.

For the AOS case, the azimuthally averaged spin–spin correlation shown in Fig. 3e exhibits a peak at low *q* that appears in a fraction of a picosecond. In this measurement, the maximum measured *q* ≈ 0.46 nm^−1^ was insufficient to determine the appearance of a Gaussian peak at higher wavevectors. Spin–spin correlation profiles at selected time instances are shown in Fig. 3f, with vertically shifted curves for the sake of clarity. Reliable fits were obtained solely by use of a Lorentzian line-shape, shown with the dashed red curves in Fig. 3f.

### Numerical simulations

**Fig. 4:**
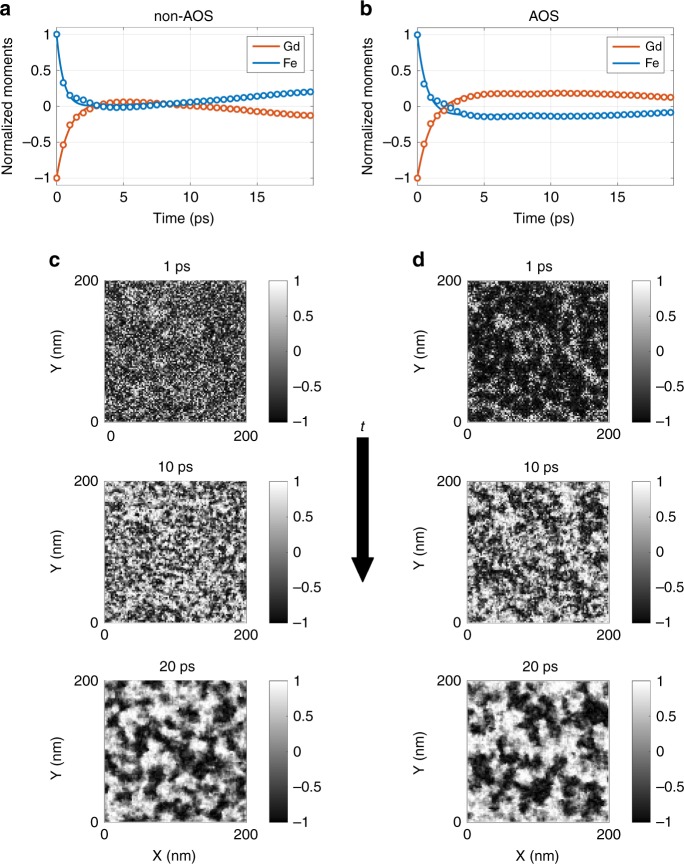
Simulated magnetisation dynamics. Normalised Gd and Fe average magnetic moments from atomistic simulations in the case of **a** non-AOS obtained with a fluence of 10.7 mJ cm^−2^, and **b** AOS obtained with a fluence of 11 mJ cm^−2^. Snapshots of the perpendicular-to-plane magnetisation at 1 ps, 10 ps, and 20 ps for the case of **c** non-AOS and **d** AOS. In both cases, the magnetisation exhibits coarsening of textures

To better understand the physical mechanisms that are most important in driving spatially inhomogeneous magnetisation dynamics after pumping, we performed atomistic simulations^40,41^. The amorphous alloy is modelled as a polycrystalline Gd and Fe–Co thin film with elemental inhomogeneity with a characteristic length of 7 nm, guided by recent experimental results^8^. The spatially averaged magnetic moments for Gd and Fe obtained with atomistic simulations are shown in Fig. 4a for the non-AOS case utilising an absorbed fluence of 10.7 mJ cm^−2^ and Fig. 4b for the AOS case utilising a similar absorbed fluence of 11 mJ cm^−2^. The atomistic simulations assume uniform heating across the thickness, and the utilised fluences are tuned to qualitatively reproduce the experimental XMCD data, cf. to Fig. 3a, b.

Snapshots of the simulated perpendicular-to-plane magnetisation evolution are shown in Fig. 4c, d for non-AOS and AOS, respectively. In both cases, coarsening of the perpendicular-to-plane magnetisation from a fine-grained randomised state is observed. The similar spatial evolution for both cases suggests that the same dynamic processes take place after ultrafast optical pumping, insofar as the magnetic moments are substantially quenched. The coarsening of the spatially varying magnetisation at such short time-scales is necessarily the result of spin-conserving nonlinear magnon interactions, whereby spatial localisation of textures rapidly minimises magnon energy^21,22^ while maintaining a quenched, average magnetisation. This is in contrast to the simple picture of field-driven growth of domains in an applied field that is operative on much longer timescales, on the order of hundreds of picoseconds^42^.

**Fig. 5:**
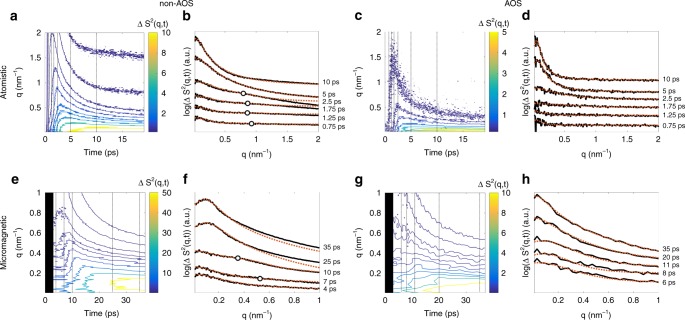
Simulated spin–spin correlation functions. **a** Contours of the azimuthally averaged spin–spin correlation function obtained from atomistic simulations in the non-AOS case. For the time instances indicated by dotted vertical lines, lineouts are shown by black curves in (**b**) and are vertically shifted for clarity. Fits using Lorentzian and Gaussian components are shown by red dashed lines. The ring radius of the Gaussian component is shown by black circles. Equivalent plots for the case of AOS are shown in panels (**c**) and (**d**). Fits to the lineouts in this case are obtained by using only a Lorentzian line-shape. For micromagnetic simulations seeded with an atomistic input at 3 ps, the azimuthally averaged spin–spin correlation function and corresponding lineouts and fits are shown in (**e**) and (**f**) for non-AOS; and (**g**) and (**h**) for AOS

To directly compare with the experimental results, the simulated spin–spin correlation function is calculated via Fourier analysis of the spatially-dependent perpendicular-to-plane magnetisation. Contours of the azimuthally averaged spin–spin correlation function in the non-AOS case are shown in Fig. 5a. Lineouts at selected time instances are shown in Fig. 5b in addition to fits of the relevant diffraction rings by a linear combination of Lorentzian and Gaussian functions centred at *q* > 0 with radius positions indicated by black circles. While the appearance of the Gaussian peak is less apparent than in the case for the data in Fig. 3d, the precise fitting of the ring radius and width was still possible, as we further demonstrate below. For the case of AOS, contours of the azimuthally averaged spin–spin correlation function are shown in Fig. 5c while selected lineouts and Lorentzian fits are shown in Fig. 5d by solid black and dashed red curves, respectively. Both cases qualitative agree with the experimental data.

To further identify the role of exchange coupling between the rare earth (Gd) and transition metal (Fe) lattices, we performed multiscale micromagnetic simulations based on the Landau–Lifshitz (LL) equation^43^ that consider an effective, homogeneous exchange stiffness, see Supplementary Note 1. The ferrimagnetic GdFeCo is modelled as a single-species ferromagnet, with an initial condition provided by the atomistic simulations at a specified delay *t*_c_ ≥ 3 ps after optical pumping. By use of this multiscale approach, we can isolate the role of the atomic-scale exchange interactions, which dominate at short times, from the longer-range exchange stiffness. Because the micromagnetic model approximates exchange dispersion as *q*^2^, spatial fluctuations should be sufficiently concentrated on small wavenumbers. However, the choice of *t*_c_ within 10 ps does not significantly change the qualitative features of the magnetisation’s coarsening (see Supplementary Note 2). As such, we only show a representative example at the shortest delay, *t*_c_ = 3 ps, at the limit of the micromagnetic approximation.

For micromagnetic simulations in the non-AOS case, the azimuthally averaged spin–spin correlation function is shown in Fig. 5e. The black area indicates the temporal range in which atomistic simulations are used to calculate the initial conditions for the micromagnetic simulations. Corresponding lineouts, along with fits by the previously described sum of Lorentzian and Gaussian functions, are shown in Fig. 5f by solid black and dashed red curves, respectively. A striking feature is that the Gaussian profile diffraction ring, identified by black circles, persists as long as 10 ps, suggesting that slower dynamics are at play in the micromagnetic approximation. After 10 ps, fits of the low-*q* diffraction ring with a Lorentzian function break down. Better fits are achieved by use of a squared Lorentzian function that exhibits a *q*^−4^-like decay. This is an artefact associated with the approximation that the magnetisation in each cell is uniform, with sharp magnetic interfaces between cells. Such a form factor is an artificial constraint in the finite-difference micromagnetic simulations that is avoided in atomistic simulations. For the case of AOS, the azimuthally averaged spin–spin correlation function is shown in Fig. 5g. Lineouts and corresponding squared Lorentzian fits are shown in Fig. 5h. The qualitative agreement to both experimental data and atomistic simulations suggests that atomic-scale exchange interactions have a limited influence on the dynamics when only a single line-shape can be fitted.

### Imprinted demagnetisation and dissociation

**Fig. 6:**
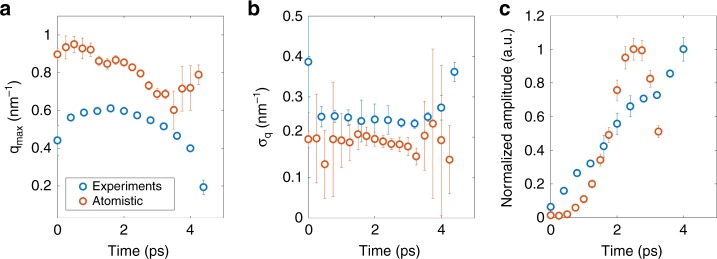
Imprinted demagnetisation and dissociation for non-AOS. Fitted parameters of the Gaussian feature from experiments (blue circles) and atomistic simulations (red circles): **a** ring radius, **b** ring width, and **c** normalised amplitude. The appearance of a pattern seeded by the material’s chemical inhomogeneity is evidenced by the relatively constant ring radius and ring width after optical pumping accompanied by a growth in the normalised amplitude. At longer times, the magnetic texture dissociates from the chemical inhomogeneities evidenced by the sudden drop of the experimental ring radius and a drop in the atomistic amplitude. Error bars represent standard deviation

The high-*q* diffraction ring in the non-AOS case appears during the optically-induced quench of the magnetic moments, indicating short-range correlations. To conclusively elucidate the physical mechanisms that drive the magnetisation dynamics at such short timescales, we analyse the fitted parameters obtained from experiments and atomistic simulations. The fitted Gaussian line-shape with ring radius *q*_max_, ring width *σ*_*q*_, and normalised ring amplitude are shown in Fig. 6a–c, respectively. Detailed fits to the experimental data are shown in Supplementary Note 3.

The blue circles are obtained from fits to experiments. For the first ≈3 ps, the XMCD data in Fig. 3a indicates that the spatially averaged Gd magnetisation is 75% quenched. At the same time, both the ring radius and the ring width are approximately constant at *q*_max_ = 0.57 ± 0.014 nm^−1^ and *σ*_*q*_ = 0.24 ± 0.002 nm^−1^, though the ring amplitude continues to increase. The ring radius is consistent with a magnetisation pattern of 2π/*q*_max_ ≈ 11 nm characteristic correlation length, similar to the ≈10 nm average chemical inhomogeneity characteristic of such amorphous GdFeCo alloys^8^. Fits to atomistic simulations shown by the red circles in Fig. 6 exhibit a similar behaviour. For the simulated fluence, the demagnetisation is almost 100% during the first 2 ps, and the average fitted ring radius and ring width are *q*_max_ = 0.88 ± 0.012 nm^−1^ and *σ*_*q*_ = 0.18 ± 0.04 nm^−1^, respectively. The corresponding correlation length of 2π/*q*_max_ ≈ 7.1 nm agrees well with the chemical correlation length used for the simulation. The diffraction ring amplitude also increases with time for the first 2 ps during the fastest part of the demagnetisation.

Both experiments and simulations provide evidence for an optically-induced spatial demagnetisation pattern that imprints on the material’s chemical inhomogeneities. The short spatial fluctuations require an atomistic description. This imprinted demagnetisation is supported by the previously identified sub-ps transfer of angular momentum between chemical species^8^.

Between ≈3 ps and ≈4.5 ps, the fitted ring radius from experiments shifts towards lower *q*. Unlike the experimental data, the high-*q* diffraction ring amplitude from atomistic simulations collapses after 3 ps, making further comparison between experiments and simulations impossible. Regardless, the shift in the ring radius for both data and simulations indicates that the magnetic system dissociates from the sample’s chemical inhomogeneity, and transitions into a state where any correlations are emergent features of the magnetisation energetics itself. We interpret these features as a collection of randomly located localised spin textures. Such a rapid dissociation is facilitated by the nonlinear attraction of high energy magnons to each other^21^. This effect is a consequence of the focusing nature of the effective nonlinear anisotropy and is well-known in other nonlinear systems such as photonics^44,45^ and Bose–Einstein condensates^46^. For this reason, we generically refer to the resulting localised textures as magnon drops^22^.

In the case of AOS, we surmise that a similar imprinted demagnetisation and subsequent dissociation processes must occur at short time scales, as suggested in ref. ^8^. However, our experimental data was not collected at the relevant wavevectors and atomistic simulations did not exhibit strong enough features to be reliably fitted.

### Magnon localisation and coalescence

The subsequent evolution of the magnetisation is quantified from the Lorentzian fits to the low-*q* diffraction ring. Details of the fitting procedure are discussed in Supplementary Note 4. The two fitted quantities of interest are the ring radius and the ring width. These quantities provide information on the size of and spatial spacing between magnon drops. A collection of randomly located magnon drops, regardless of their spatial spacing, constitute random telegraph noise that results in a Lorentzian line-shape centred at *q* = 0 whose ring width is inversely proportional to the average magnon drop size. However, because overlapping magnon drops compose a single feature, a finite ring radius inversely proportional to the magnon drops’ spatial spacing ensues. These spectral features are similar to those observed in X-ray scattering experiments of a molecular liquid–liquid transition where hard-core-like repulsive interactions between so-called locally favoured structures is invoked^47^.

The absence of harmonics indicates that the magnon drops’ size distribution dominates the scattering: a periodic array of identically sized magnon drops would consist of harmonic rings by virtue of a Fourier series decomposition whose harmonic-dependent coefficients would encode the magnon drops’ size and profile. Deviations of the lattice periodicity would result only in the rings’ spectral broadening.

**Fig. 7:**
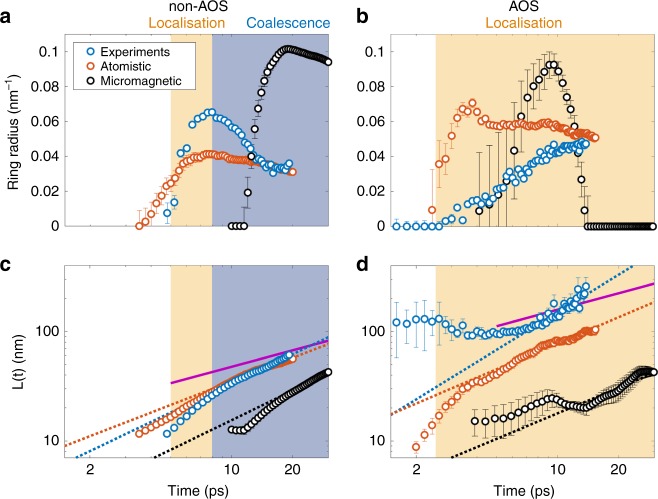
Magnon localisation and coalescence. The ring radius of the Lorentzian feature is shown for **a** non-AOS and **b** AOS. Time is plotted in logarithmic scale. Symbols represent experimental (blue circles), atomistic (red circles), and micromagnetic (black circles) data. The average magnon drop diameter *L*(*t*) is shown in log–log scale for **c** non-AOS and **d** AOS. Dotted lines with corresponding colour code are power-law fits. The magenta solid line indicates the Lifshitz–Cahn–Allen power law. While *L*(*t*) increases according to a power law for all cases, the expanding ring radius is a signature of magnon localisation, indicated by the gold-shaded area. The shrinking ring radius observed only for the non-AOS case is the signature of magnon coalescence, indicated by the blue-shaded area. Error bars represent standard deviation

The evolution of the fitted ring radius is shown in Fig. 7a, b for non-AOS and AOS cases, respectively. The evolution of the average magnon drop diameter *L* = 2*π*/Δ*q*, where Δ*q* is the ring width, is shown in Fig. 7c, d for non-AOS and AOS cases. Blue, red, and black circles correspond to, respectively, experiments, atomistic simulations, and micromagnetic simulations.

In the experimental non-AOS case, a ring with a non-zero radius appears after 5 ps. The ring radius increases for the first 3 ps, indicating that the average spatial spacing is decreasing as magnon drops continue to nucleate. A maximum ring radius of 0.0626 ± 0.0011 nm^−1^ is observed at 8 ps, corresponding to an average spatial spacing of ≈100 ± 11 nm. During the same temporal window, the average magnon drop diameter increases from ≈10 to ≈20 nm. The observation of an expanding ring radius accompanied by the growth of magnon drop diameters defines the magnon localisation process. During this stage, an initially sparse collection of magnon drops becomes close-packed due to the continual nucleation of magnon drops.

At longer times, the ring radius drops and appears to reach a plateau at 0.0334 ± 0.0016 nm^−1^ that corresponds to an average spacing of ≈188 ± 9 nm. However, *L* continues to increase approximately following a power law growth. This behaviour is consistent with the growth of the already present magnon drops via merger and break-up as well as nonlinear attraction of thermal magnons, so that the average spatial spacing increases. We refer to this dynamical process as magnon coalescence and is characterised by the shrinking of the scattered ring radius and its width.

The fits to the atomistic data in the non-AOS case return a qualitatively similar behaviour for both the ring radius and *L*. Micromagnetic simulations exhibit delayed development of the low-*q* diffraction ring, with an onset of a non-zero ring radius at ≈10 ps. Such delayed dynamics result from the micromagnetic magnon dispersion proportional to *q*^2^, an approximation that is only valid at long wavelengths, as opposed to the more accurate 1-cos(*qa*) form associated with quantum mechanical exchange in a periodic lattice with lattice constant *a*. Consequently, upon transference of the spatial magnetisation distribution from the atomistic to the micromagnetic simulations, magnons with wavenumbers approaching the Brillouin zone boundary convey an artificially inflated amount of thermal energy into the spin system. This artificially inflated spin temperature commensurately increases the time required to form magnon drops of a similar size to those obtained via the more accurate atomistic simulations. Despite this caveat, the qualitative features of the low-*q* ring obtained micromagnetically agree with the occurrence of magnon localisation and magnon coalescence.

For the AOS case, Fig. 7b, the low-*q* ring radius for the experimental data is non-zero after ≈2.5 ps. The ring radius monotonically increases to a maximum of ≈0.047 ± 0.001 nm^−1^ at the longest experimental delay time of 20 ps, corresponding to a minimum average spacing of ≈133 ± 2 nm. The associated evolution of *L*, Fig. 7d, exhibits a fixed value of ≈100 nm for ≈7 ps, after which *L* gradually increases to ≈250 nm out to the longest delay times. Viewed together with the evolution of the average spatial spacing, the process of AOS appears to be one in which switching is mediated by an ever decreasing spacing between drops, due to monotonically increasing density of magnon drops. In other words, only magnon localisation is operative for AOS, in a manner consistent with the eventual switching of the macroscopic magnetisation. This is in clear contrast to the case of non-AOS, where the magnon localisation is arrested and gives way to magnon coalescence.

Atomistic simulations in the AOS case yield a much more rapid increase in the ring radius on a time scale of 4 ps, followed by a slow reduction until the radius is close to that of the experimental data at the longest delay. Micromagnetic simulations exhibit a similar increase in the ring radius between 4 and 10 ps, at which point the maximum ring radius is ≈0.09 nm^−1^. However, there is a rapid collapse of the ring radius after 10 ps, such that the spatial spacing completely diverges at 11 ps. The failure of both the atomistic and micromagnetic models to quantitatively reproduce the evolution of the ring radius in the AOS case suggests that there remains important non-equilibrium physics that affect the rapid magnetisation dynamics in amorphous alloys, which are not contained in either our atomistic or micromagnetic simulations.

Despite differences in ring radii for both AOS and non-AOS, the similar monotonic increase of *L* for both experiments and simulations with time after 4 ps, indicates that the necessary physics to describe the growth of magnon drops at ps time scales are properly captured by the models. More importantly, the fact that micromagnetic simulations can qualitatively describe the evolution of *L* beyond 10 ps indicates that nothing more than exchange stiffness and uniaxial anisotropy are necessary to drive the growth of magnon drops.

Power law fits to *L* are shown in Fig. 7c, d by colour coded dashed lines that utilise the fitting function $L\left( t \right) = bt^a$. The resultant fitting parameters are listed in Table 1. We find exponents in the range 0.71 ≤ *a* ≤ 1.14 for all cases. Fits to experimental data obtained at different fluences for both Gd and Fe yield exponents of similar values (see Supplementary Note 5). Taking into account exponents obtained from experiments and simulations, we obtain an average exponent of *a* = 0.82 ± 0.04.

**Table 1:** Fitted parameters for the power law *L*(*t*) = *bt*^*a*^

		Experiment	Atomistic simulations	Micromagnetic simulations
Non-AOS	*a*	0.88 ± 0.01	0.71 ± 0.01	0.89 ± 0.007
	*b* (nm/ps)	4.36 ± 0.11	6.71 ± 0.25	1.95 ± 0.05
AOS	*a*	1.14 ± 0.15	0.78 ± 0.03	0.77 ± 0.01
	*b* (nm/ps)	10.84 ± 3.97	12.68 ± 0.79	2.99 ± 0.15

Domain growth in 2nd order phase kinetics in a non-conservative system is typically modelled with the Lifshitz–Cahn–Allen (LCA) theory, which postulates that domain wall velocity is linearly proportional to the local curvature of the phase interface. This leads to power-law growth of phase-ordered domains with an exponent of 1/2^26,48,49^, confirmed in 2D simulations, e.g., refs. ^50^^,^^51^. Solid magenta lines in Fig. 7c, d show exemplary LCA behaviour. However, LCA assumes domain growth proceeds by progression through a continuous series of intermediate, energetically favoured meta-stable states via short-range interactions. As such, the applicability of LCA is questionable in the case of magnon drops, where spins at the magnon drop perimeter are necessarily dynamic, and where long-range spin interactions can be mediated by both nonlocal dipole fields^29^ and the hydrodynamic flow of angular momentum via exchange^23,24,52,53^, all of which would tend to decouple the domain growth rate from the phase boundary local curvature.

### Magnon drop dynamics via exchange flows of spin current

Analysis of the magnetisation dynamics from a micromagnetic perspective can shed light onto the origins of how *L* grows during magnon coalescence. Interactions between magnon drops can be quantified and visualised in terms of the long-range transport of angular momentum arising from the noncollinear magnetisation^54^. Utilising a hydrodynamic formulation^23,24,52,53^, the transfer of angular momentum is represented by EFSCs. EFSCs can be expressed as an equivalent charge current density that is 100% spin polarised in the perpendicular-to-plane direction^23,24^.

**Fig. 8:**
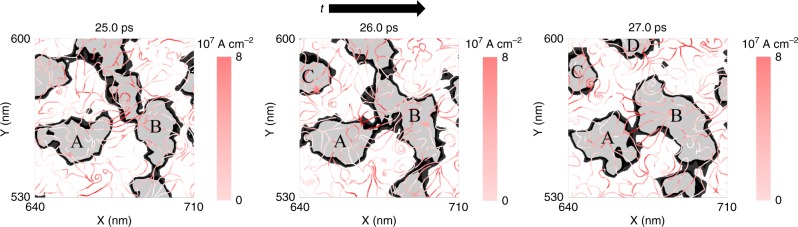
Large transfer of angular momentum. The snapshots show the evolution of magnon drops, including merger and break-up. The black areas represent magnon drop perimeters (|*m*_*z*_| < 0.2) and the white and grey areas indicate that the perpendicular-to-plane magnetisation is preferentially parallel or antiparallel to the applied field. The pink-shaded curves represent EFSCs expressed as equivalent 100% spin polarised charge current. The streamlines indicate the instantaneous transfer of perpendicular-to-plane angular momentum

Examples of EFSCs mediating magnon drop interactions in micromagnetic simulations are shown in Fig. 8 by three snapshots spanning a 2 ps time interval. The magnon drops’ perimeters, where the perpendicular-to-plane magnetisation is less than 0.2, are represented as solid black areas. The white and grey background indicate areas where the perpendicular-to-plane magnetisation is preferentially parallel (*m*_*z*_ > 0.2) or anti-parallel (*m*_*z*_ < -0.2) to the applied field, respectively. The grey areas are nascent magnon drops in the early evolution of the phase coarsening process. The pink-shaded streamlines represent the flow of angular momentum quantified by EFSCs. We find that EFSCs with an equivalent charge current density on the order of 10^7^ A cm^−2^ can persist for many 10’s of ps after optical pumping. Such magnitudes are similar to those used for magnetisation switching via spin transfer torque^55^. While the EFSCs are spatially nonuniform and highly turbulent, they can effectively deform the magnon drops’ perimeters, causing both breathing and rotating^25.56^. Furthermore, the EFSCs mediate long-range interactions between magnon drops that result in both mergers and break-ups^25^. An example of a merger is observed between the leftmost and central magnon drops, labelled A and B, respectively. At 25 ps, large-magnitude EFSCs flow between the magnon drops, so that a torque is exerted at the perimeters. At 26 and 27 ps, the perimeters merge into a single drop B and continue to transfer angular momentum. Examples of break-up are observed at the top of the central magnon drop, where EFSCs transfer angular momentum away from the magnon drop. As a result, the top-left and top magnon drops, labelled C and D, break up at 26 and 27 ps, respectively.

## Discussion

While our experimental and numerical results focus on the first 20 ps evolution of the magnetisation in GdFeCo alloys, these dynamics shed light onto the nonlinear magnon processes that drive coarsening while the spin system equilibrates.

The qualitative agreement between experiments and multiscale simulations demonstrates that current models incorporate the most important physical effects responsible for the nonlinear magnon processes identified here. From a theoretical perspective, this agreement implies that appropriate scaling of the equation of motion can be used to describe other magnetic materials insofar as they exhibit PMA and a net magnetisation at equilibrium. In other words, the magnon processes described here are universal for PMA ferromagnets and ferrimagnets. To substantiate this claim, we performed additional atomistic simulations for a chemically homogeneous GdFeCo (see Supplementary Note 6). The evolution of the spatially varying magnetisation is consistent with a magnon coalescence process at times greater than 5 ps. Because there are no inhomogeneities to seed a magnetisation pattern during demagnetisation, we conclude that magnon localisation and coalescence in PMA magnets are indeed general processes that are independent of the material’s structure, although the details of the spatial evolution process can be affected by the presence of chemical inhomogeneities. In addition, micromagnetic simulations in the magnon coalescence regime exhibit qualitatively similar evolution of both the ring radius and the average magnon drop diameter *L* for different sample thicknesses, initial state of spatial magnetisation, and variation of other micromagnetic parameters (see Supplementary Note 7).

The hydrodynamic formulation of magnetisation dynamics provides a valuable tool to understand the long-time magnetisation evolution. For example, the nucleation of topological defects after fast quench^36,37^ represents an interesting possibility in the context of optically-induced applications. In the hydrodynamic formulation, topological defects are evidenced by curved trajectories in the EFSCs^23^. However, an accurate calculation of the topological number for a spatially localised defect requires a uniform magnetisation surrounding the defect^57^. In other words, defects must be sparsely located. We surmise that understanding the evolution of EFSCs from tens to hundreds of picoseconds timescales, where dissipative processes are operable, will lead to a better understanding of the dynamic evolution of topological defects upon ultrafast optical pumping.

Based on our study, we speculate that desired magnetisation states may be stabilised by nanopatterning magnetic materials to take advantage of both sub-picosecond seeded magnetisation states and EFSCs, even for crystalline materials. For example, a close-packed spatially periodic patterning may favour a like-wise close-packed magnetisation pattern during localisation to induce AOS. Conversely, sparse engineered defects may serve as pinning potentials to stabilise topological defects.

## Methods

### Experiments

The GdFeCo samples were fabricated on 100 nm thick Si_3_N_4_ membranes by magnetron sputtering. A 5 nm seed layer of Si_3_N_4_ was first grown on the membrane followed by the Gd_0.24_Fe_0.665_Co_0.095_ film, which was then capped with 20 nm of Si_3_N_4_. X-ray measurements were conducted at the SXR hutch of the Linac Coherent Light Source^58^. The X-ray energy was selected to be resonant with the Fe L_3_ resonance edge at 707 eV or the Gd M_5_ resonance edge at 1185 eV with a 0.5 eV bandwidth and a pulse duration of 80 fs. The X-ray pulses were circularly polarised at the Fe L_3_ and Gd M_5_ edges by using the XMCD in magnetised Fe and GdFe films, respectively placed upstream of the experiment. A degree of polarisation was 85% at the Fe L_3_ edge and 79% at the Gd M_5_ edge. Measurements were made in transmission geometry with X-rays incident along the sample normal. An in-vacuum electromagnet was used to apply a field of 0.5 T perpendicular to the GdFeCo film. The diffracted X-rays were collected with a p–n charge-coupled device (pnCCD) two-dimensional detector placed behind the sample. A hole in the centre of the detector allowed the transmitted beam to propagate to a second detector used to collect the transmitted X-ray beam. The experiment was conducted in an optical pump—X-ray probe geometry. Optical pulses of 1.55 eV and 50 fs duration were incident on the sample in a near collinear geometry. The delay between the optical and X-ray pulses was achieved using a mechanical delay line, where the delay was continuously varied. X-ray-optical jitter was monitored and removed from the experimental data using an upstream cross-correlation arrival monitor^59^.

### Atomistic simulations

A model system of a GdFe ferrimagnet was developed to perform numerical simulations of the atomistic spin dynamics after femtosecond laser excitation. The inhomogeneous microstructure is generated by specifying random seed points representing areas of segregation of the Gd from the alloy, leading to 15–30% higher local Gd concentration. These regions are interpolated using a Gaussian with a standard deviation of 5 nm, representing the scale of the segregation. Due to low packing of the seed points, the characteristic length of the spatial variations is approximately 7 nm. An atomistic level simulation model is used to properly describe the ferrimagnetic ordering of the atomic moments with Heisenberg exchange^40^. The energy of the system is described by the spin Hamiltonian

1 $${\cal{H}} = - \mathop {\sum }\limits_{i < j} J_{ij}{\mathbf{S}}_i \cdot {\mathbf{S}}_j - \mathop {\sum }\limits_i k_u\left( {S_i^z} \right)^2,$$

where the spin **S**_*i*_ is a unit vector describing the local spin direction. *J*_*ij*_ is the exchange integral, which we limit to nearest neighbour interactions, and *k*_*u*_ is the anisotropy constant. Time-dependent spin dynamics are governed by the Landau–Lifshitz equation at the atomistic level

2 $${\partial} _t{\mathbf{S}}_i = - \frac{\gamma }{{\left( {1 + {\alpha} ^2} \right)}}[{\mathbf{S}}_i \times {\mathbf{B}}_{{\mathrm{eff}}}^i + \alpha {\mathbf{S}}_i \times ({\mathbf{S}}_i \times {\mathbf{B}}_{{\mathrm{eff}}}^i)],$$

where *γ* is the gyromagnetic ratio and *α* = 0.01 is the Gilbert damping factor that can be used in the Landau-Lifshitz form when α << 1. The on-site effective induction can be derived from the spin Hamiltonian with the local field augmented by a random field to model the interactions between the spin and the heat bath

3 $${\mathbf{B}}_{{\mathrm{eff}}}^i = - \frac{1}{{{\mu} _i}}\;\frac{{\partial {\cal{H}}}}{{\partial {\mathbf{S}}_i}} + {\varsigma} _i,$$

where the second term $\varsigma _i$ is a stochastic thermal field due to the interaction of the conduction electrons with the local spins, and *μ*_*i*_ is the local (atomic) spin magnetic moment. The stochastic thermal field is assumed to have Gaussian statistics and satisfies

4 $$\left\langle {\varsigma _{i,{\mathrm{a}}}\left( t \right)\varsigma _{j,{\mathrm{b}}}\left( {t^\prime } \right)} \right\rangle = \delta _{ij}\delta _{ab}\left( {t - t^\prime } \right)2\alpha _ik_{\mathrm{B}}T_{\mathrm{e}}/(\gamma _i\mu _i),$$

5 $$\left\langle {\varsigma _{i,{\mathrm{a}}}(t)} \right\rangle = 0,$$

where *k*_B_ is the Boltzmann constant and *T* is the temperature. We incorporate the rapid change in thermal energy of a system under the influence of a femtosecond laser pulse. The spin system is coupled to the electron temperature, *T*_e_, which is calculated using the two-temperature model^60^ with the free electron approximation for the electrons

6 $$T_eC_{\mathrm{e}}\frac{{dT_{\mathrm{e}}}}{{dt}} = - G_{{\mathrm{el}}}\left( {T_{\mathrm{l}} - T_{\mathrm{e}}} \right) + P\left( t \right),$$

7 $$C_{\mathrm{l}}\frac{{dT_{\mathrm{l}}}}{{dt}} = - G_{{\mathrm{el}}}\left( {T_{\mathrm{e}} - T_{\mathrm{l}}} \right),$$

where *C*_*e*_ = 225 J m^−3^ K^−1^, *C*_1_ = 3.1 × 10^6^ J m^−3^ K^−1^, *G*_el_ = 2.5 × 10^17^ W m^−3^ K^−1^, and *P*(t) models the temperature from a single Gaussian pulse into the electronic system. The pulse has a width of 50 fs.

We use Heun numerical integration scheme to integrate the stochastic equation of motion with time-varying temperature^41^. We use $\mu _{{\mathrm{F}}_{\mathrm{e}}} = 1.92\mu _B$ as an effective magnetic moment containing the contribution of Fe and Co and we set $\mu _{{\mathrm{G}}_{\mathrm{d}}} = 7.63\mu _B$ for the Gd sites, where *μ*_*B*_ is Bohr’s magneton. The standard parameters of the exchange coupling constants are used: $J_{{\mathrm{Fe - Fe}}} = 4.526 \times 10^{ - 21}$ J per link, $J_{{\mathrm{Gd - Gd}}} = 1.26 \times 10^{ - 21}$ J per link, and $J_{{\mathrm{Fe}} - {\mathrm{Gd}}} = - 1.09 \times 10^{ - 21}$ J per link. We assume a uniaxial anisotropy energy of 8.07246 × 10^−24^ J per atom. The numerical simulations are conducted using the VAMPIRE software package^41^. The simulation volumes were 200 nm × 200 nm × 2 nm and 1000 nm × 1000 nm × 2 nm.

### Multiscale micromagnetic simulations

Micromagnetic simulations were performed with the graphic processing unit (GPU) package MuMax3^61^ that solves the Landau–Lifshitz equation for a ferromagnet

8 $$\partial _t{\bf{m}} = - \gamma \mu _0\left[ {{\bf{m}} \times {\bf{B}}_{{\mathrm{eff}}} + \alpha {\bf{m}} \times {\bf{m}} \times {\bf{B}}_{{\mathrm{eff}}}} \right],$$

where *μ*_0_ is the vacuum permeability, **m** is the magnetisation vector normalised to the saturation magnetisation, and **B**_eff_ is an effective induction that includes the required physical terms to model a ferromagnetic material. Here, we included exchange, nonlocal dipole, uniaxial anisotropy, and external fields. The exchange interaction in the micromagnetic approximation takes the form of a Laplacian scaled by the exchange length, *λ*_ex_. In MuMax3, the Laplacian is numerically resolved by a 4th order central finite difference scheme, i.e., each micromagnetic cell is subject to exchange interaction due to itself and two neighbouring cells in each dimension. We ran our simulations on NVIDIA GPU units K20M, K40, K80, and P100. Due to the coarse resolution of micromagnetic simulations, we utilise approximately cubic cells of size 2 nm × 2 nm × *δ*, where *δ* = *D*/2 ^*N*^ and the factor *N* is chosen to take advantage of the GPU spectral calculations such that *δ* < *λ*_ex_ ≈ 5 nm and *D* is the physical thicknesses equal to 30 or 20 nm for the non-AOS or AOS cases, respectively. The lateral simulation area was determined from atomistic simulations and the full thickness for each case was achieved upon the atomistic observation that the magnetisation is approximately homogeneous across the thickness at *t* ≥ 3 ps. The coarse micromagnetic discretization allows for a significant speed up in the computations. Note that the size of the cells only impacts the stability and accuracy of the numerical algorithm while the physics can only be interpreted in the framework of the continuum Landau–Lifshitz equation, i.e., long-wavelength features relative to the exchange length. We set the software to solve Eq. (8) with an adaptive-step, 4th order Runge–Kutta time integration method. Periodic boundary conditions (PBCs) were imposed along the film’s plane. For both dynamical behaviours we used the equilibrium magnetic parameters: saturation magnetisation *M*_S_ = 47170.6 A m^−1^, anisotropy constant *k*_u_ = 31127.228 J m^−2^, exchange constant *A* = 1 pJ m^−1^, and *α* = 0.01. The value for *A* was numerically found to best match the atomistic, average perpendicular magnetisation evolution (see Supplementary Note 1).

### Change in the spin–spin correlation function

Experimentally, the change in the spin–spin correlation function, Δ*S*^2^(*q,t*), was obtained from the scattered intensities of circularly polarised X-rays. For this, the scattering intensities are added to obtain

9 $$S^2\left( {q,t} \right) + C^2\left( q \right) = \frac{{I_ + \left( {q,t} \right) + I_ - \left( {q,t} \right)}}{2},$$

where *I*_+_(*q*,*t*) and *I*_−_(*q*,*t*) are the time-dependent scattered intensities obtained with right-handed and left-handed circularly polarised light, *S*^2^(*q,t*) is the spin contribution to the intensity, and *C*^2^(*q*) is the charge contribution to the intensity. Because the charge contribution is time-independent for the used pump fluences, the spin–spin correlation function can be isolated as

10 $$\Delta S^2\left( {q,t} \right) = S^2\left( {q,t} \right) - \langle S^2\left( {q,t < 0} \right)\rangle,$$

where the background was subtracted by averaging the data collected at times before the optical pulse irradiated the sample.

To compare the data with simulations, the spin–spin correlation function for both atomistic and micromagnetic simulations was determined by computing a two-dimensional fast Fourier transform (FFT) of the perpendicular magnetisation for each layer as a function of time. To minimise error, the FFTs obtained for each layer at a given time were averaged. Since PBCs were used for simulations, a window function was not necessary.

### Exchange flow spin currents

In the dispersive hydrodynamic formulation of magnetisation dynamics^23,24^, the normalised magnetisation vector **m** = (*m*_*x*_, *m*_*y*_, *m*_*z*_) in Eq. (8) can be cast in hydrodynamic variables by the canonical transformation

11 $$\begin{array}{*{20}{c}} {n = m_z}, & {{\bf{u}} = - \nabla {\mathrm{arctan}}\left[ {m_y/m_x} \right]} \end{array},$$

where *n* is the spin density and **u** is the fluid velocity. For the case of conservative dynamics, *α* = 0 in Eq. (8), the dispersive hydrodynamic equations are

12 $$\partial _tn = \nabla \cdot \left[ {\left( {1 - n^2} \right)\mathbf{u}} \right],$$

13 $$\partial _tu = - \nabla \left[ {\left( {1 - \left| {\bf{u}} \right|^2} \right)n} \right] - \nabla \left[ {\frac{{\Delta n}}{{1 - n^2}} + \frac{{n\left| {\nabla n} \right|^2}}{{\left( {1 - n^2} \right)^2}}} \right] - \nabla h_0,$$

expressed in dimensionless space, time, and field scaled by, respectively $\sqrt {\left| {H_{\mathrm{k}}/M_{\mathrm{S}} - 1} \right|} \lambda _{{\mathrm{ex}}}^{ - 1}$, $\gamma \mu _o\left| {H_{\mathrm{k}} - M_{\mathrm{S}}} \right|$, and $M_{\mathrm{S}}^{ - 1}$, where the anisotropy field is given by $H_{\mathrm{k}} = 2k_{\mathrm{u}}/\left( {\mu _oM_{\mathrm{S}}} \right)$, and *h*_0_ is a dimensionless field applied normal to the plane. The spin density flux in Eq. (13) is identified as the EFSC in hydrodynamic variables. To establish a clear comparison to spin currents obtained by charge-to-spin transduction, the EFSC are expressed as a 100% spin polarised charge current density in units of A m^−2^ by^24^

14 $${\bf{J}}_{\mathrm{S}} = - \frac{{2e}}{\hbar }\mu _0M_{\mathrm{S}}^2\lambda _{{\mathrm{ex}}}\left( {\frac{{H_{\mathrm{k}}}}{{M_{\mathrm{S}}}} - 1} \right)^{ - 1/2}\left( {1 - n^2} \right){\bf{u}},$$

We note that the factor (1 − *n*^2^) leads to maximum EFSC for a given **u** when the magnetisation is in the plane. For this reason, the magnon drop perimeters are primarily subject to EFSCs.

## Supplementary information

Supplementary Information

Description of Additional Supplementary Files

Supplementary Movie 1

Supplementary Movie 2

## Data Availability

The data that supports the findings of this study are available from the corresponding author upon reasonable request.

## Code availability

MuMax3 is open source software and available from http://mumax.github.io. VAMPIRE 5 is open source software and available from https://vampire.york.ac.uk. The data fitting procedure and calculation of EFSC were custom written in MATLAB and are available from the corresponding and leading author upon reasonable request.
