# Supplementary material for: Spin-current-mediated rapid magnon localisation and coalescence after ultrafast optical pumping of ferrimagnetic alloys
Source: Nat Commun. 2019 Apr 15;10:1756. doi: 10.1038/s41467-019-09577-0 (PMC6465265; doi:10.1038/s41467-019-09577-0)
Supplement: Supplementary file 1 — Supplementary Information [file 41467_2019_9577_MOESM1_ESM.pdf]

Spin-current-mediated rapid magnon localisation and coalescence after ultrafast optical pumping of ferrimagnetic alloys

Iacocca et al.

Supplementary Note 1. Micromagnetic exchange constant: average atomistic and micromagnetic dynamics

To obtain a multiscale model, the micromagnetic parameters for the GdFeCo alloy were chosen to match atomistic simulations. The saturation magnetisation, anisotropy constant, and damping can be directly obtained from atomistic simulations. The exchange constant is challenging to obtain because it requires an average on the element and spatially dependent Heisenberg exchange. The addition of inhomogeneity adds complexity to the spatial average calculation that leads to an imprecise determination of a micromagnetic exchange constant. To circumvent this problem, we utilised a numerical approach to estimate the micromagnetic exchange constant based on the qualitative behaviour of the average perpendicular magnetisation, $<m_z>$.

The goal was to choose an exchange constant such that the temporal evolution of $<m_z>$ calculated from micromagnetic simulations utilising atomistic magnetisation states as inputs at different times was both self-consistent, i.e., followed the same qualitative evolution, and consistent with atomistic simulations. The results obtained with an exchange constant A = 1 pJ m^{-1} are shown in the Supplementary Figure 1. Utilising atomistic spatial magnetisation as initial conditions at and after 3 ps, the micromagnetic simulations exhibit a slow evolution of $<m_z>$ that is qualitatively consistent between the different micromagnetic simulations, shown by circles, and agrees with the effective perpendicular magnetisation obtained from atomistic simulations, shown by a dashed black curve.

For the atomistic spatial magnetisation at 1 ps and 2 ps, a stark disagreement is observed. This occurs because of the predominantly switched average magnetisation at short times after the demagnetisation event. Note that while the magnetic moments partially quench after 3 ps, the large magnetic moment of Gd relative to Fe leads to an average switched magnetisation before 3 ps in the multiscale modelling. Micromagnetic simulations model a ferromagnet and, consequently, has no available physical mechanism to recover the short-range order based on the antiferromagnetic Gd-Fe exchange interaction. For the atomistic input magnetisation at 1 ps, the dominantly switched magnetisation translates into a large anisotropy energy that strives to relax the magnetisation towards the negative pole, i.e., $m_z = -1$. For the atomistic input magnetisation at 2 ps, the magnetisation is close to zero. While for ferrimagnets this implies average compensated moments, in micromagnetic simulations this implies that the saturation magnetisation is negligibly small and, consequently, the dynamics are extremely slow.

We emphasize that the choice of the exchange constant described here is not critical to model the qualitative features of magnon coalescence nor impacts the conclusions drawn in the main text.

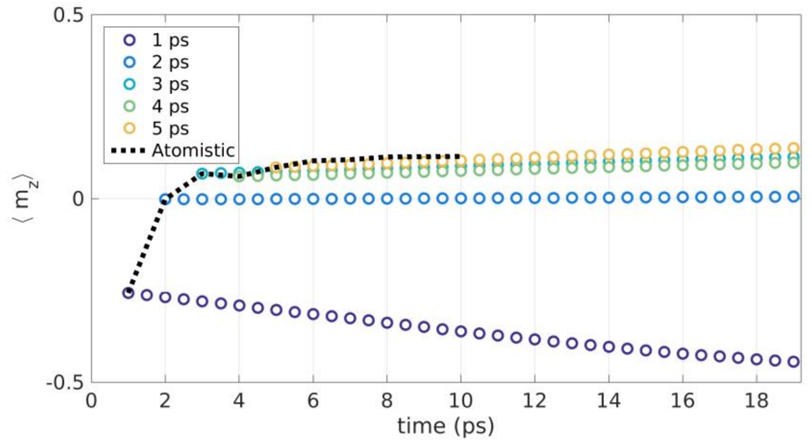

Supplementary Figure 1**. Average magnetisation evolution from simulations.** Micromagnetic evolution of the average perpendicular magnetisation utilising atomistic spatial magnetisation as inputs at 1 ps, 2 ps, 3 ps, 4 ps, and 5 ps, shown by circles. The evolution of the effective perpendicular magnetisation from atomistic simulations is shown by a dashed black curve.

Supplementary Note 2. Multiscale simulations for the non-AOS case as a function of t_c

The micromagnetic magnon drop diameter growth presented in the main text was obtained by initialising the micromagnetic simulations with the atomistic spatial magnetisation at 3 ps. However, as shown in the Supplementary Figure 1, micromagnetic simulations exhibit a self-consistent behaviour utilising atomistic magnetisation states as inputs after 3 ps. The average magnon drop diameter growth calculated from Lorentzian fits to the azimuthally averaged spin-spin correlation function from micromagnetic simulations initialised with atomistic magnetisation states at times 3 ps to 9 ps in steps of 1 ps is shown in the Supplementary Figure 2. Despite a quantitative difference at short timescales (between 10 and 15 ps), the power law growth converges to a similar slope after 25 ps, indicating that the multiscale simulations are accurately resolved.

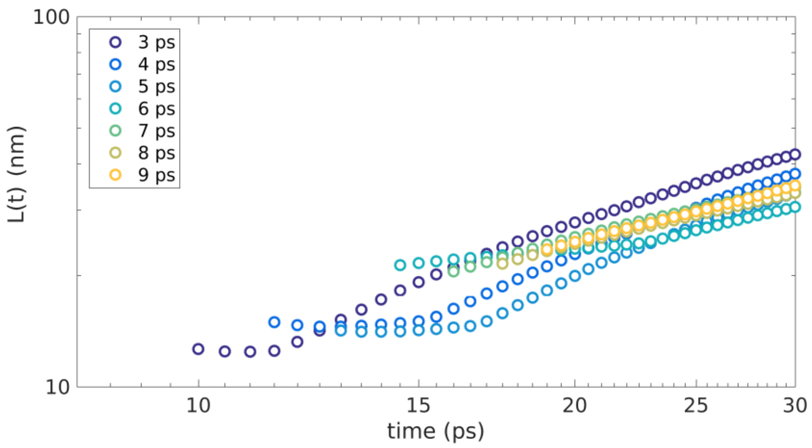

Supplementary Figure 2**. Multiscale magnon drops' diameter growth for the non-AOS case.** Average magnon drop diameter growth calculated from Lorentzian fits to the spin-spin correlation function obtained from micromagnetic simulations initialised with atomistic magnetisation states. Error bars are not shown here for clarity.

Supplementary Note 3. Short-time evolution of Gaussian feature

A Gaussian feature that corresponds to the magnetisation pattern seeded by the material chemical inhomogeneity is observed in the spin-spin correlation function for Gd. In the Supplementary Figure 3a, the data obtained for Gd in the non-AOS case is shown as artificially shifted solid black curves from 0 ps (bottom lineout) to 4.8 ps (upper lineout). Fits with a Lorentzian and a Gaussian component are shown by dashed red curves. The ring radius of the Gaussian component in time is shown by black circles. Whereas a Gaussian component can be fitted at 0 ps with some accuracy (when the sample is at thermal equilibrium), the feature is clearly seen only at the first measured delay after the femtosecond pulse. The corresponding evolution of the fitted Gaussian component is shown in the Supplementary Figure 3b.

We note that fits obtained by utilising a Lorentzian line-shape return similar metrics. An example of a Lorentzian line-shape fit at t = 0.8 ps is shown in the Supplementary Figure 3c by a dashed blue curve. The fit is very similar to that obtained with a Gaussian line-shape shown by a dashed red curve. However, we find as a general trend, that a Gaussian line-shape returns smaller errors in the fitted quantities than a Lorentzian line-shape.

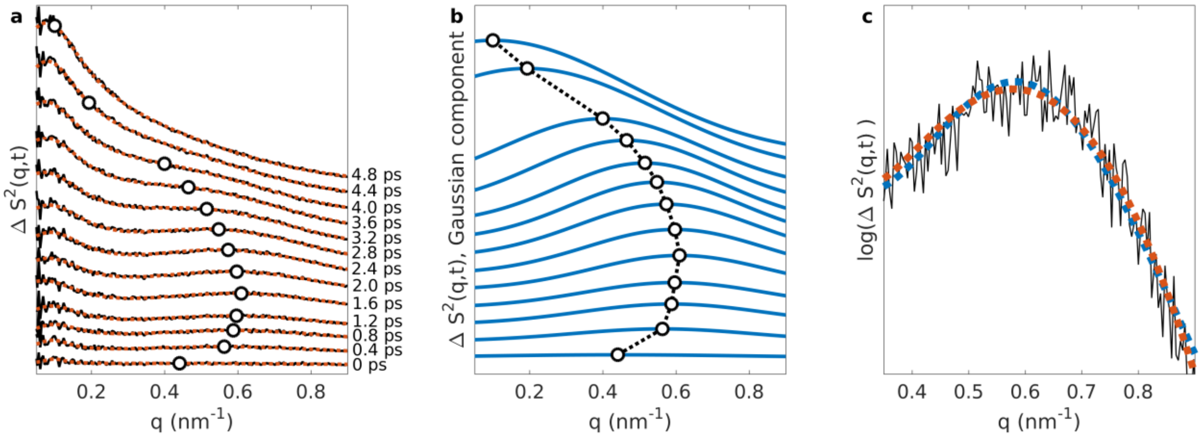

Supplementary Figure 3**. Short time evolution of Gaussian component in the spin-spin correlation function. a** Lineouts of the spin-spin correlation function measured for Gd between 0 and 4.8 ps at a fluence of 3.91 mJ cm^{-2} are shown by shifted solid black curves. Fits with Lorentzian and Gaussian components are shown by dashed red curves. **b** Gaussian component of the fitted lineouts. The ring radius of the Gaussian component is shown by black circles in both panels. **c** Comparison of fits utilising a Lorentzian (dashed blue curve) and a Gaussian (dashed red curve) component.

Supplementary Note 4. Fitting procedure for experimental data during magnon coalescence

The ring observed during magnon coalescence is located close to the smallest resolved q. To reliably fit the ring radius, we use a Lorentzian function of the form

$$f_{\mathrm{L}}(q \geq q_0) = \frac{A}{(q-p)^2+\gamma^2} + B \quad (1)$$

where A is a factor proportional to the Lorentzian's integral, p is the ring radius, γ is the full-width at half-maximum, and B is a background. We also consider a fitting threshold, q_0. We vary q_0 from the experimental minimum of 0.024 nm^{-1} to 0.06 nm^{-1}. The goal is to determine if the fitted peak is a feature in the data or a fitting artefact due to the low signal-to-noise ratio close to the experimental minimum q. For this, we compute the mean deviation of the fitted ring radius at q_0 relative to the ring radius fitted for the experimental minimum, q_{min}.

The results for the case of non-AOS are summarised in the Supplementary Figure 4. We identify three regions. In R1, the mean deviation exhibits an initial increase because of the low signal-to-noise ratio close to q_{min}. In R2, the mean deviation exhibits a minimum that is indicative of a robust ring radius fit. In other words, the small mean deviation is a result of random fluctuations about the fitted ring radius with a negligible bias. In R3, the mean deviation increases, indicating that the fitted ring radius is not accurate. In fact, $p < q_0$ in R3, so that only data in the Lorentzian tail is used for fitting. Therefore, we identify $q_0 = 0.048$ nm^{-1} as the optimum fitting threshold. The same analysis was performed for the experimental data in the case of AOS.

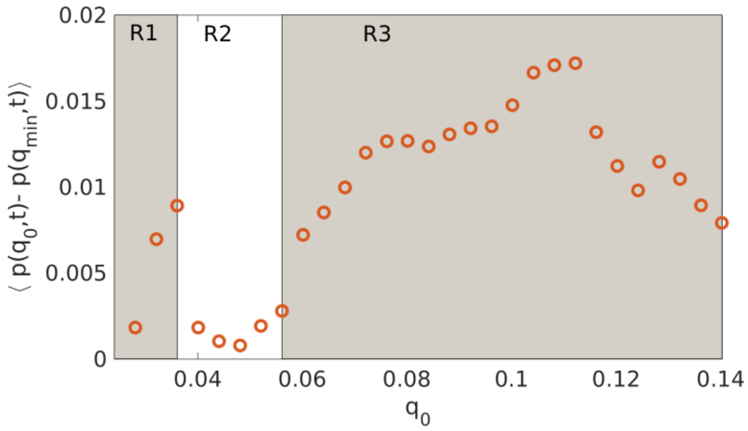

Supplementary Figure 4. Mean deviation of fitted ring radius. The minimum deviation in R2 indicates that the ring radius is reliably fitted with small error.

Supplementary Note 5. Magnon localisation and coalescence for non-AOS: Gd and Fe

Partial quench of the magnetic moments is observed in our GdFeCo alloys for a range of fluences. X-ray scattering is measured simultaneously for Gd and Fe because of the technique's element specificity. For both elements and the absorbed laser fluences of 3.91 mJ cm^{-2}, 2.79 mJ cm^{-2}, and 1.39 mJ cm^{-2}, the contours of the azimuthally averaged spin-spin correlation function shown in the top row of the Supplementary Figure 5 exhibit similar qualitative features. The data for Fe has a lower signal-to-noise ratio but reliable fits to Lorentzian line-shapes are achieved after 5 ps. The fitted peak position and calculated average magnon drop diameter L for each case is shown in the bottom row of the Supplementary Figure 5. Power-law fits can be obtained at long times for all cases, with parameters shown in each panel.

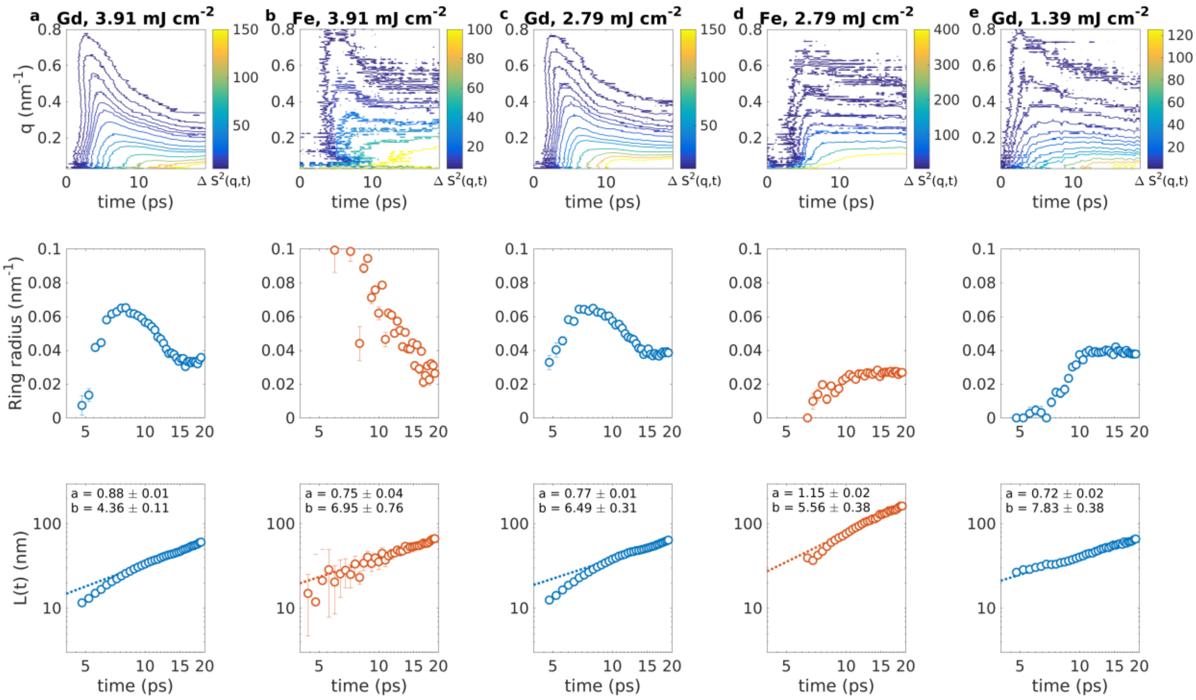

Supplementary Figure 5. **Experimental data for Gd and Fe at several fluences.** Top row: contours of the azimuthally averaged spin-spin correlation function for the indicated element and fluence, namely, **a** Gd at 3.91 mJ cm^{-2}, **b** Fe at 3.91 mJ cm^{-2}, **c** Gd at 2.79 mJ cm^{-2}, **d** Fe at 2.79 mJ cm^{-2}, and **e** Gd at 1.39 mJ cm^{-2}. Bottom row: $L(t)$ calculated from Lorentzian fits to the azimuthally averaged spin-spin correlation function. Error bars represent standard deviation.

Supplementary Note 6. Atomistic simulations for chemically homogeneous GdFeCo in the non-AOS case

We perform atomistic simulations for crystalline or chemically homogeneous GdFeCo. Partial quench of magnetic moments is achieved for a narrow window of fluences. While quantitative differences are expected, we find a similar evolution of the spatial magnetisation, as shown in the Supplementary Figure 6a and b.

Fits to the resulting spin-spin correlation function during magnon coalescence provides evidence that magnon localisation occurs at much shorter timescales. Because of the lack of chemical inhomogeneities, a Gaussian peak cannot be fitted but a finite ring radius is observed already at 0.5 ps, although with large error bars. These results demonstrate that chemical inhomogeneities impact the magnon localisation process, but long-range magnetic order is nonetheless recovered by the nucleation and subsequent evolution of localised textures.

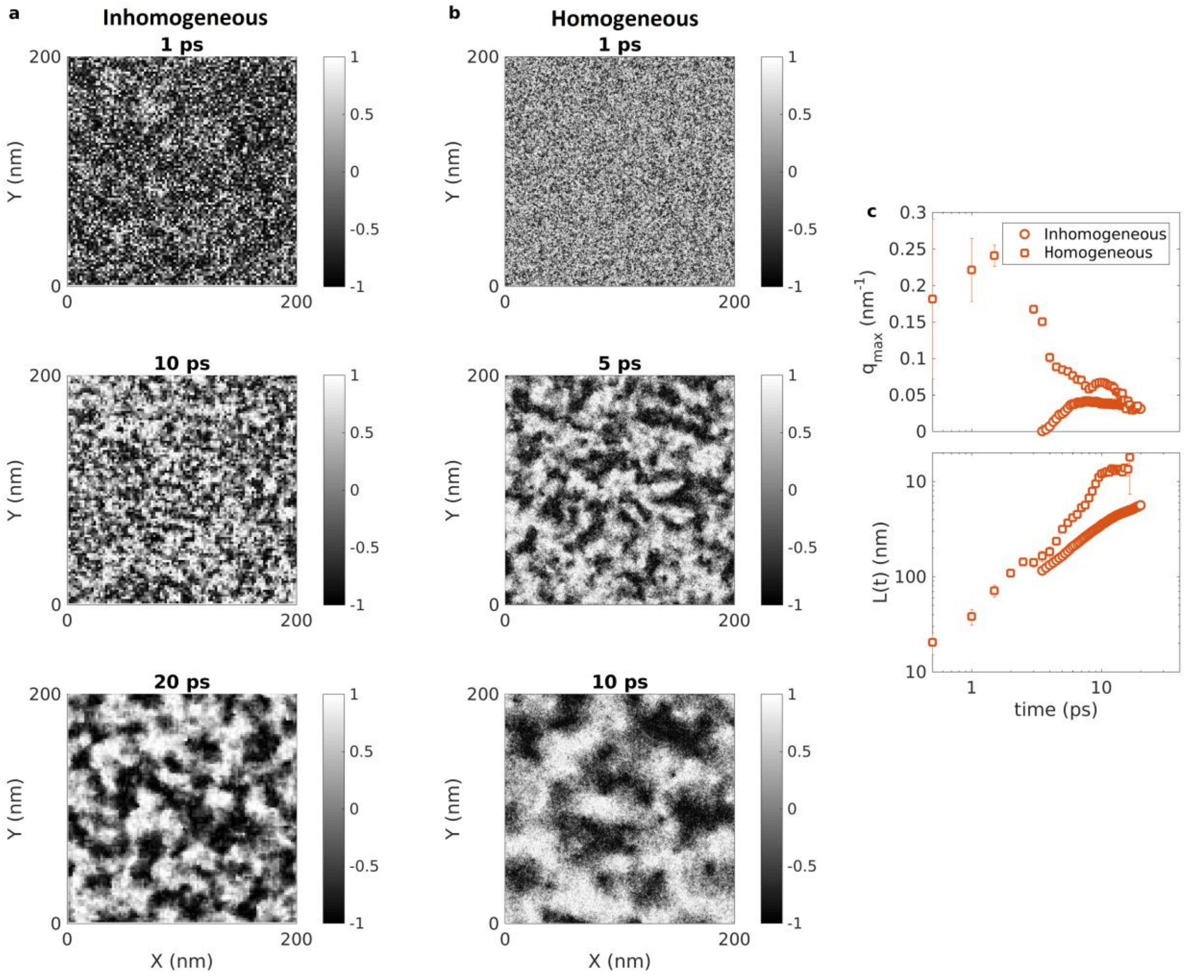

Supplementary Figure 6. Atomistic simulations for inhomogeneous and homogeneous GdFeCo in the non-AOS case. The left column **a** shows the spatial magnetisation evolution including chemical inhomogeneities and is reported in the main text, Figure 4a. The right column **b** shows the spatial magnetisation evolution considering a homogeneous material. A comparison between the fitted ring radius and average magnon drop diameter scale during magnon coalescence is shown in **c**. Error bars represent standard deviation.

Supplementary Note 7. Universality of magnon coalescence during non-AOS: micromagnetic simulations

In the main text, we demonstrated that magnon coalescence is primarily driven by exchange and uniaxial anisotropy. These terms are captured by the micromagnetic approximation. An important consequence is that magnon coalescence should be observed regardless of the initial state of the spatial magnetisation and the material parameters.

To explore the above conjecture, we have performed additional micromagnetic simulations and the fitted ring radius and linewidth during magnon coalescence for all cases are reported in the Supplementary Figure 7.

First, we initialise the micromagnetic simulation with a fully random magnetisation, i.e., both the polar and azimuthal angle of the magnetisation vector are uniformly distributed. The fitted ring radius and average magnon drop diameters L are shown by squares in the

Supplementary Figure 7a. The fits to the multiscale simulation are shown by circles and correspond to the results shown in the main text Figure 7a and c. Remarkably, we observe a similar evolution of the ring radius, emphasizing that the nonlinear magnon interactions resulting in localised textures are important.

Second, we repeat the multiscale simulations with varying film thicknesses. The evolution is essentially identical for all cases, as shown in the Supplementary Figure 7b by colour-coded circles as specified in the legend. These simulations indicate that the nonlocal dipole field depending on the film thickness is not important at the short time scales investigated here. Therefore, the evolution of magnon drops is mainly the result of a balance between exchange and anisotropy.

Finally, we perform simulations initialised with a random magnetisation distribution and material parameters of Co/Ni multilayers. This is a well-known ferromagnetic material that exhibits perpendicular magnetic anisotropy and we use the parameters M_S =716.2 kA m^{-1}, k_u = 447 kJ m^{-2}, A = 15 pJ m^{-1} and α = 0.04. The results are shown in the Supplementary Figure 7c. While the quantitative results differ significantly, we observe a qualitatively similar behaviour of both the ring radius and L. The anisotropy of Co/Ni is an order of magnitude larger than that of GdFeCo and may be responsible for the sub-picosecond magnon localisation. However, L exhibits a similar evolution. The reason behind such a similar evolution may suggest a universal behaviour, but further theoretical work is required to demonstrate or disprove this statement.

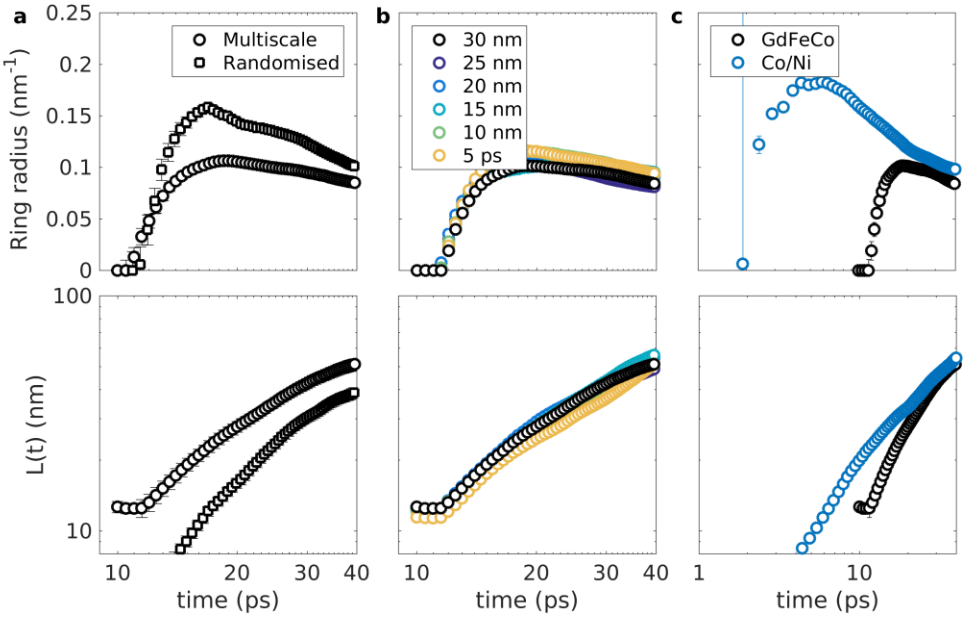

Supplementary Figure 7**. Fits during magnon coalescence from micromagnetic simulations. a** GdFeCo parameters with randomly distributed magnetisation. **b** Multiscale GdFeCo simulations with several film thicknesses. **c** Co/Ni parameters with randomly distributed magnetisation. Error bars represent standard deviation
