# Supplementary material for: Spin-current-mediated rapid magnon localisation and coalescence after ultrafast optical pumping of ferrimagnetic alloys
Source: Nat Commun. 2019 Apr 15;10:1756. doi: 10.1038/s41467-019-09577-0 (PMC6465265; doi:10.1038/s41467-019-09577-0)
Supplement: Supplementary file 2 — Description of Additional Supplementary Files [file 41467_2019_9577_MOESM2_ESM.pdf]

Description of Additional Supplementary Files

File name: Supplementary Movie 1
Description: Evolution of the perpendicular-to-plane magnetisation from micromagnetic simulations for the non-AOS case. The interval 3 ps to 37.5 ps is shown.

File name: Supplementary Movie 2
Description: Evolution of the magnetisation from a hydrodynamic perspective. The movie displays a zoomed area of the micromagnetic simulations for the non-AOS case. The pink-shaded streamlines represent exchange flow spin currents colour-coded by an equivalent 100% spin polarised charge current. The black areas represent the magnon drop's perimeters (perpendicular-to-plane magnetisation magnitude less than 0.2), white areas perpendicular-to-plane magnetisation parallel to the field, and the grey areas perpendicular-to-plane magnetisation antiparallel to the field. Three snapshots are shown in Figure 8 in the main text. The interval 3 ps to 37.5 ps is shown.
